# Evidence of a Distinctive Enantioselective Binding Mode for the Photoinduced Radical Cyclization of α-Chloroamides in Ene-Reductases

**DOI:** 10.1021/acscatal.3c03934

**Published:** 2023-11-10

**Authors:** Matteo Capone, Gianluca Dell’Orletta, Bryce T. Nicholls, Gregory D. Scholes, Todd K. Hyster, Massimiliano Aschi, Isabella Daidone

**Affiliations:** † Department of Physical and Chemical Sciences, University of L’Aquila, via Vetoio (Coppito 1), L’Aquila 67010, Italy; ‡ Department of Chemistry and Chemical Biology, Cornell University, Ithaca, New York 14853, United States; § Department of Chemistry, Frick Laboratory, Princeton University, Princeton, New Jersey 08544, United States

**Keywords:** enantioselectivity, biocatalysis, photocatalysis, asymmetric synthesis, radical cyclization, π-facial

## Abstract

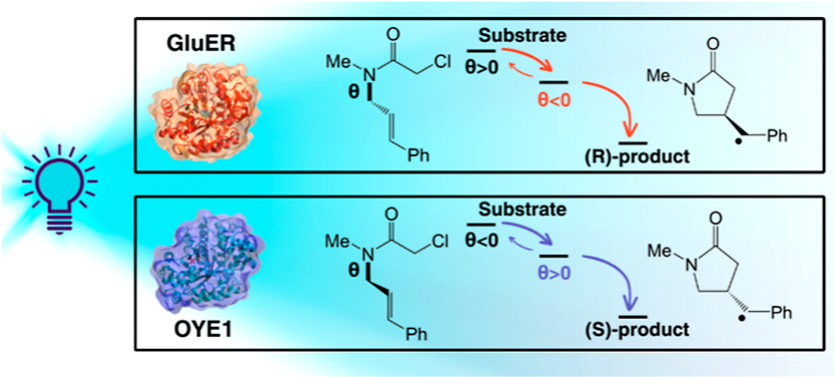

We demonstrate here through molecular simulations and mutational studies the origin of the enantioselectivity in the photoinduced radical cyclization of α-chloroacetamides catalyzed by ene-reductases, in particular the *Gluconobacter oxidans* ene-reductase and the Old Yellow Enzyme 1, which show opposite enantioselectivity. Our results reveal that neither the π-facial selectivity model nor a protein-induced selective stabilization of the transition states is able to explain the enantioselectivity of the radical cyclization in the studied flavoenzymes. We propose a new enantioinduction scenario according to which enantioselectivity is indeed controlled by transition-state stability; however, the relative stability of the prochiral transition states is not determined by direct interaction with the protein but is rather dependent on an inherent degree of freedom within the substrate itself. This intrinsic degree of freedom, distinct from the traditional π-facial exposure mode, can be controlled by the substrate conformational selection upon binding to the enzyme.

## Introduction

Enzymes serve as exceptional catalysts for asymmetric synthesis, thanks to their remarkable ability to achieve precise stereocontrol in complex chemical reactions. Their applications range from small-scale synthesis to large-scale industrial manufacturing.^1−5^ By repurposing naturally occurring enzymatic machinery, the utilization of non-natural chemistries holds the potential to broaden the range of reactions in biocatalysis.^6−12^

An important class of reactions enabling the synthesis of structural motifs found in agrochemical and pharmaceutical compounds^13^ relies on the selective coupling of electrophilic radicals and alkenes. However, the development of catalytic strategies to achieve stereoselectivity in this type of transformation has proven to be challenging.^14^ This difficulty primarily stems from the requirement to maintain a close association between radical species and chiral catalysts during the step that determines stereoselectivity.

**Figure 1:**
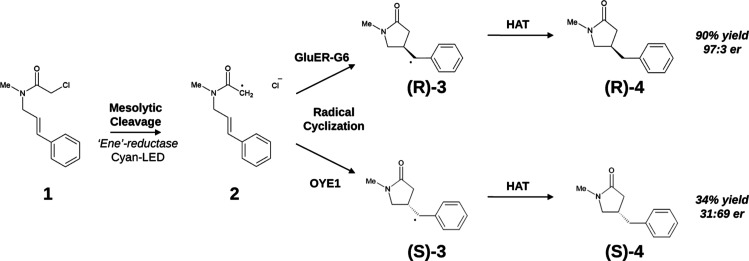
Photoinduced enantioselective radical cyclization of α-chloroacetamide-(**1**) to γ-lactam-(**4**) with EREDs. As demonstrated in previous works,^15,18^ the first step of the reaction involves photoinduced mesolytic cleavage of the C–Cl bond to form the radical substrate **2** followed by radical cyclization to the exocyclic radical **3**. The reaction is terminated via HAT from flavin to the exocyclic radical. The yield and enantiomeric excess in GluER-G6^18^ and OYE1^15^ are reported on the right.

Recently, a biocatalytic strategy utilizing the photoactivation of flavoenzymes for Csp^3^–Csp^3^ bond-forming reactions has been proposed.^15−19^ The radical cyclization of α-chloroamides to obtain β-stereogenic lactams, which are prevalent motifs in medically significant compounds,^20^ was addressed. In particular, the efficacy of flavin-dependent “ene”-reductases (EREDs) in achieving the desired transformation was successfully demonstrated,^15,17,18^ and the general mechanism of the photoinduced reaction was determined based on isotope incorporation experiments, transient absorption spectroscopy, and UV–vis spectroscopy.^15,18^ It has been demonstrated that the substrate and the flavin hydroquinone (FMN_*hq*_) cofactor form an electron donor–acceptor (EDA) complex within the enzyme active site and that irradiation under cyan light excites the EDA complex to a charge transfer state. The flavin hydroquinone cofactor acts as a single electron reductant, leading to the initial formation of a radical intermediate through mesolytic cleavage of the C–Cl bond.^15^ Then, after radical cyclization to an exocyclic radical, the reaction is terminated through a hydrogen atom transfer (HAT) from the neutral flavin semiquinone (FMN_*sq*_^•^) to reach the product and oxidized flavin (FMN_*ox*_) (see Figure 1).^15^ These findings highlight the suitability of EREDs in hosting radical intermediates and their capacity to regulate the stereochemical outcome of radical reactions.

In order to propel the progress of this recently developed enzymatic photoactivated biocatalysis, it is crucial to deepen the comprehension of the reaction mechanism and the underlying factors contributing to the enzyme’s control over stereoselectivity. Unveiling the origin of this distinct mode of enantioinduction remains an unexplored area that warrants further investigation. So far, the stereocontrol of numerous enzymatic olefin functionalization reactions has been explained by π-facial selectivity models.^21,22^ These models rely on the differentiation between the two prochiral π-faces due to the constrained rotation of the olefin within the complex.^21−25^ Additionally, the role of selective stabilization of prochiral transition states has been demonstrated to be crucial for a more accurate understanding of enzymatic stereoselectivity.^23,26−28^ This was shown to be particularly important when the reactive functional group of the substrate, such as an olefin, has limited interactions with the protein scaffold and displays flexibility within the enzyme–substrate complex. In such a case, substrate-binding models proved ineffective, and a focus on transition states became crucial for describing the origin of enzymatic stereocontrol.^29^

Herein, we use computational methodologies to explore the origin of the enantioselectivity in the photoinduced asymmetric radical cyclization of α-chloroacetamide-(**1**) to the γ-lactam-(**4**) shown in Figure 1 within two different EREDs, which provide opposite enantiomeric excess: the triple mutant T36A-K317M-Y343F of *Gluconobacter oxidans* ene-reductase (GluER-G6), which favors the **R** enantiomer, and the Old Yellow Enzyme 1 (OYE1), which favors the **S** enantiomer. The corresponding yield and enantiomeric excess are listed in Figure 1. To this aim, we employ a multiscale methodology based on quantum-mechanical (QM) calculations, molecular dynamics (MD) simulation, and the perturbed matrix method (PMM).^30^ The combined MD-QM-PMM approach has proven its effectiveness in various applications, such as computing redox potentials,^31−33^ simulating photoinduced charge transfer processes,^33−35^ and time-resolved spectroscopic observable.^36^ By means of this approach, the dynamical effects of the complex protein environment can be taken into account in the calculation of the free energy variations associated with the cyclization process. Certainly, it is widely acknowledged that reactions occurring in enzymes’ active sites are deeply influenced by the complex dynamics of their surrounding environment,^37,38^ which also includes the solvent.^39^

We begin with the investigation of GluER-G6 and then compare the results with OYE1. First, we identified the most promising binding poses and characterized the most relevant interactions of substrate **1** with the active-site residues by means of classical MD. Subsequently, we examined the conformational flexibility of the olefin in the radical intermediate **2**, which forms after the light-driven exit of the Cl^−^ anion, within the enzyme to identify the most relevant degrees of freedom possibly affecting the radical cyclization step. Starting from the most representative configurations, the energy profiles for the cyclization to the R- and S-radical products **3** were calculated in the gas phase and the selectivity-determining radical cyclization transition states were identified. Finally, the corresponding free energy profiles within the protein were calculated by means of the MD-PMM approach. A novel mechanism, distinct from those previously reported for the functionalization of alkenes, is proposed.

## Results

### Identification of the Substrate Binding Pose in GluER-G6 through MD Simulation

**Figure 2:**
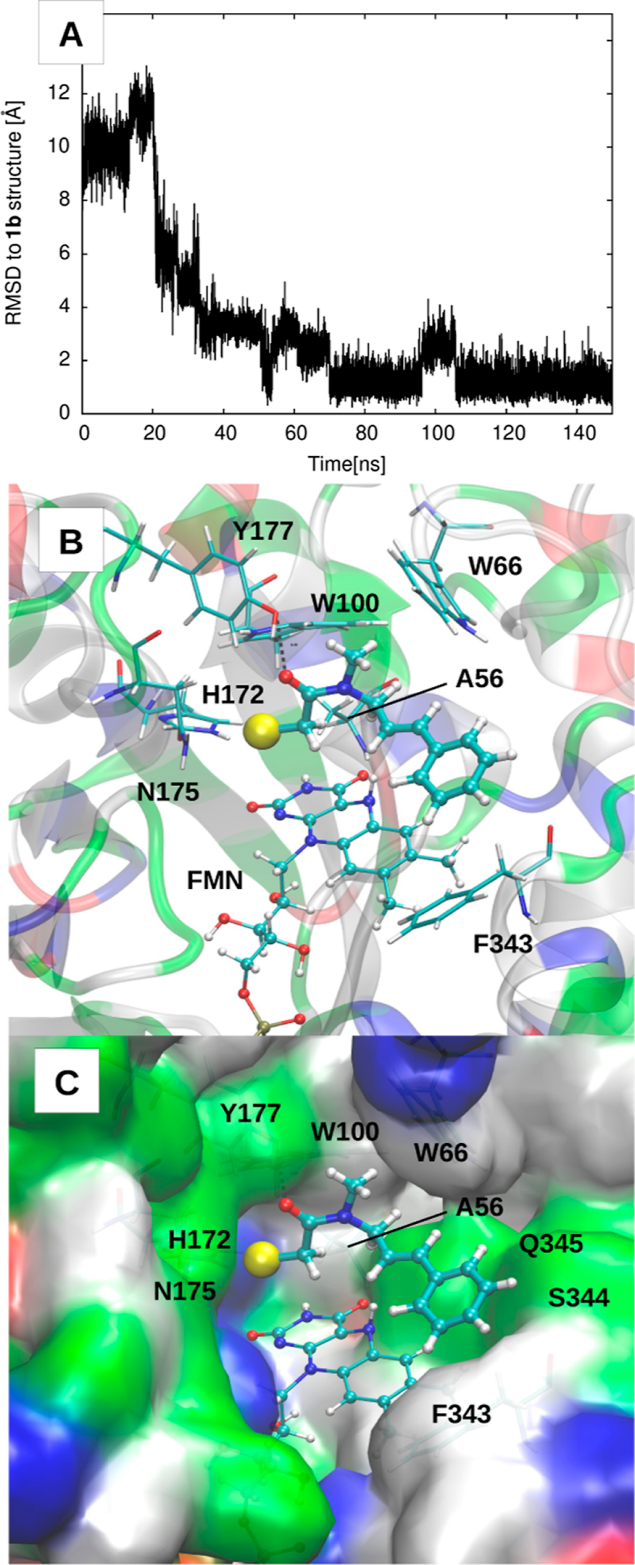
(A) Root mean square deviation of the substrate with respect to conformer **1b** (see caption to Figure 3) along the first 150 ns long MD trajectory of GluER-G6. (B) Representative configuration of the most populated EDA complex sampled along the MD simulations of GluER-G6. The stable HB between the carbonyl group of the substrate and the side chain of Y177 is highlighted. The Cl atom is shown in yellow. (C) Space-filling representation of the active-site residues to highlight the steric effect.

We performed an initial 150 ns-long MD simulation of the complex between the GluER-G6 mutant (GluER-T36A-K317M-Y343F) and substrate **1**. This simulation was started from the best binding pose obtained by rigid docking with Vina.^40^ Interestingly, after around 50 ns, the substrate changed its configuration in the binding site, and the new complex remained stable for the remaining 100 ns (see Figure 2A). We then generated two new 100 ns-long trajectories starting from complex structures extracted from the first simulation. Also in these two simulations, the binding pose remained stable. For further analysis, all the configurations generated in the three simulations were used, with the exclusion of the first 50 ns of the first trajectory.

A representative configuration of the binding site is reported in Figure 2 (panels B and C), in which the main interactions between the substrate and the active-site residues are highlighted. The α-chloroacetamide group is hosted in a pocket composed of residues A56, W66, W100, H172, N175, Y177, and R261; the carbonyl group of the substrate is involved in a stable (population higher than 90%) hydrogen bond (HB) with the side chain of Y177 (see panel B of Figure 2). Furthermore, the α-CH2 group is tightly packed within the −CH3 group of A56, the side chain of W100, and the isoalloxazine ring of the flavin, while the *N*-methyl group is less tightly bound, partly interacting with W66 and partly exposed to the solvent. The Cl atom occupies a subcavity formed by H172 and N175. The styrene moiety is packed onto the isoalloxazine ring of the flavin and is inserted into a pocket composed of residues F343, S344, Q345, and W66 (see panel C of Figure 2).

**Figure 3:**
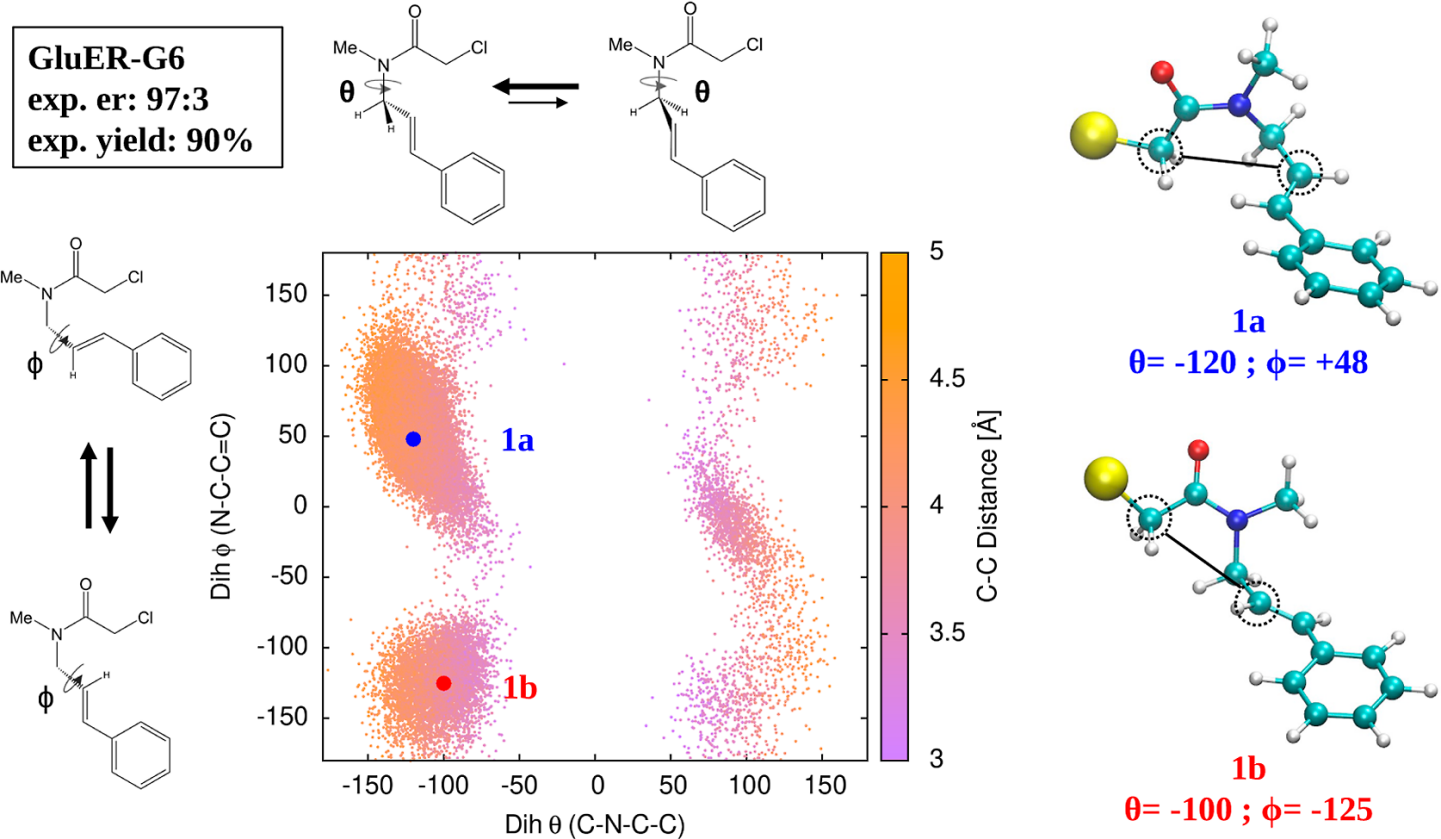
2D distribution of the θ and ϕ dihedral angles over the MD simulations of GluER-G6. θ is defined by atoms C–N–C–C and accounts for the rotation around the N–C(allyl) bond; ϕ is defined by atoms N–C–C–C and accounts for the rotation around the C(allyl)–C(vinyl) bond. The two main basins at negative θ values comprise the majority of the population (90% of the conformers). The color scale represents the carbon–carbon distance (in Å) of the vinyl and the α-acetamide carbons (C(vinyl)-C(α-acetamide)). The two most populated conformations (**1a** and **1b**) are shown on the right.

To analyze the internal flexibility of the substrate, we considered as collective variables the two most flexible dihedral angles, i.e., the one defined by atoms C–N–C–C (θ) and accounting for the rotation around the N–C(allyl) bond and the other defined by atoms N–C–C–C (ϕ) and accounting for the rotation around the C(allyl)–C(vinyl) bond, as shown in Figure 3. The bidimensional distribution of the θ/ϕ angles sampled along the MD simulations is reported in the same figure (Figure 3). In all conformers, the allyl group and the carbonyl are mainly anticlinal (θ within 90 and 150° or within −90 and −150°), while a very high conformational flexibility of the N-allyl group is observed, with ϕ spanning the whole accessible range. The flexibility associated with the ϕ angle in the enzyme–substrate complex is due to the lack of sterically bulky residues around the N-allyl. Two main basins can be identified at negative θ values, comprising by far the majority of the population (90% of the conformers). Representative substrate-conformers extracted from the two most populated clusters (**1a** with θ = −120 and ϕ = +48 and **1b** with θ = −100 and ϕ = −125) are also shown.

### Gas-Phase Radical Cyclization Energy Profiles

In what follows, we study the radical cyclization process in the absence of the protein. To this aim, representative structures of the starting α-radical intermediate **2** need to be chosen. Starting from the most representative configurations of the substrate in complex with the enzyme, i.e., structures **1a** and **1b**, the chlorine ion was removed and the generated α-radical intermediates were optimized at the quantum-chemical level (see the Computational and Experimental Methods section for details) keeping the θ and ϕ dihedrals constrained (a comparison between unconstrained and constrained optimizations is provided in the Supporting Information). The choice of this procedure is justified by the following three pieces of evidence: (i) experimentally, it was found that the photoinduced mesolytic cleavage of the C–Cl bond, which leads to the formation of the α-radical intermediate **2** (see Figure 1), is very fast (of the order of 1 ps);^18^ thus, the θ/ϕ dihedral angles are expected to undergo only minor changes; (ii) classical MD simulations of the α-radical intermediate **2** in complex with the enzyme (details of the simulations are provided in the Supporting Information) show a θ/ϕ dihedral angles distribution very similar to the one of substrate **1** in complex with the enzyme (see the Supporting Information); (iii) in a previous work in which a similar substrate was studied in complex with the enzyme P450, it was shown that the substrate and the corresponding α-radical intermediate possess similar θ/ϕ dihedral angle distributions.

**Figure 4:**
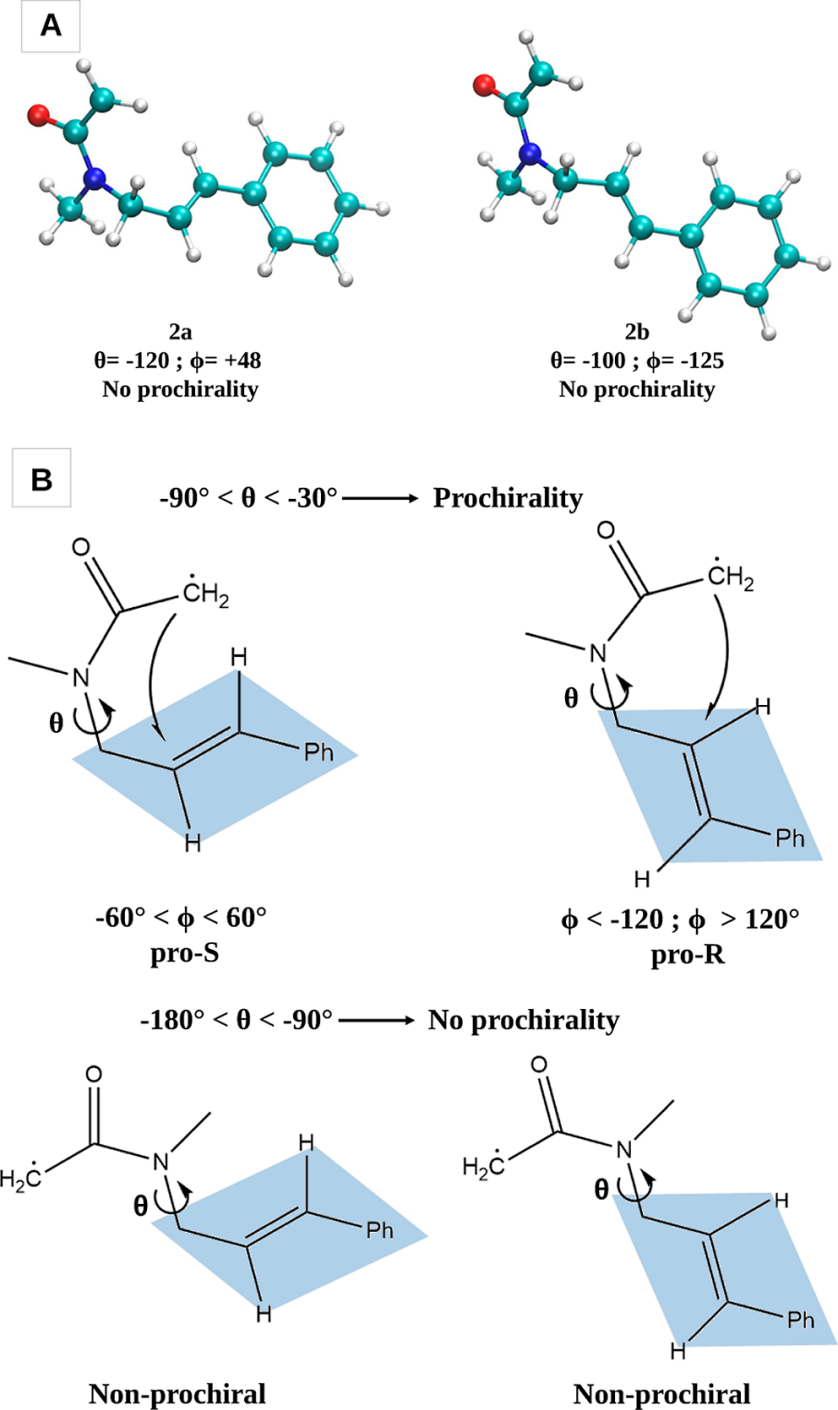
(A) Starting structures of the α-radical intermediates, **2a** and **2b**, used for the calculations of the cyclization profiles reported in Figure 5. (B) Schematic representation of the dependence of the potential prochirality of the α-radical intermediate on the θ/ϕ dihedral angles for negative θ values. The mirror scenario is valid for conformers with positive θ values.

The two optimized α-radical intermediate structures (**2a** and **2b**) are shown in Figure 4A. In principle, the prochirality of a given structure can be assessed on the base of the ϕ angle, which determines whether the (Re)-face or the (Si)-face of the C=C double bond is exposed to the α-acetamide moiety, thus potentially conferring to the α-carbonyl radical intermediate either the S- or R-prochirality, respectively. However, this holds true only under the condition that the exposure of the π-face is persistent (i.e., if the ϕ angle is not highly flexible) or if the acetamide and the allyl groups are almost periplanar (i.e., θ is in the range 0 ± 30°); in the studied system, neither of the two statements holds true because θ is not in the periplanar range and the N-allyl group is highly flexible. When the allyl group and the carbonyl are instead either synclinal (θ within −30 and −90°) or anticlinal (θ within −90 and −150°), the prochirality strongly depends on the θ angle, as schematized in Figure 4B: if −90° < θ < −30°, then the radical is either pro-S (−60° < ϕ < 60°) or pro-R (ϕ < −120°; ϕ > 120°); otherwise, no prochirality can be assigned. In our case, both the **2a** and **2b** structures are clearly nonprochiral, being the acetamide and the allyl groups anticlinal.

**Figure 5:**
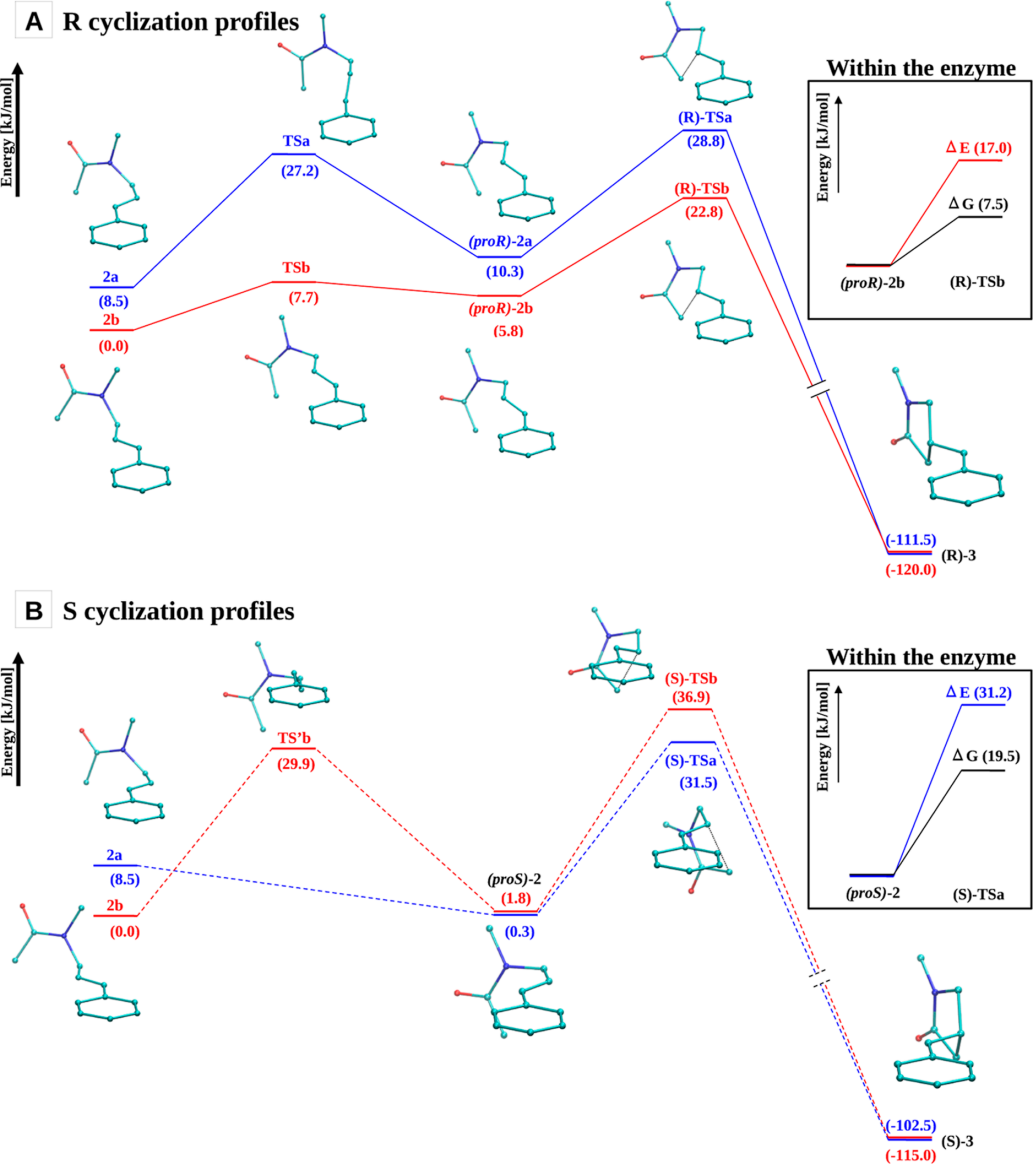
Cyclization energy profiles. Energy of reactants, possible intermediates, transition states, and products starting from the substrate radical **2a** (blue) and **2b** (red) along the **R** profiles [**2a** → **(R)-3** and **2b** → **(R)-3]**, panel A, and the **S** profiles [**2a** → **(S)-3** and **2b** → **(S)-3]**, panel B. The energies are reported with respect to the energy of the reactant **2b**. The corresponding activation free energies within the enzyme calculated with the MD-PMM approach and associated with the profiles showing the lowest cyclization barrier (i.e., the red and blue profiles in panel A and panel B, respectively) are reported in the insets. The error in the Δ*G* values is ±4 kJ/mol and is calculated as the mean (i.e., averaged over all states of all profiles) standard error, as obtained by dividing the productive MD simulations into three sets.

Starting from each of these structures, the following energy profiles for the radical cyclization process to the R- and S-radical products [i.e., **(R)-3** and **(S)-3**, respectively] were calculated using the nudged elastic band (NEB) method (see the Computational and Experimental Methods section): **2a** → **(R)-3** and **2b** → **(R)-3** (the **R** profiles) and **2a** → **(S)-3** and **2b** → **(S)-3** (the **S** profiles). The corresponding energy values for the reactants, possible intermediates, transition states, and products are reported in Figure 5 (the **R** profiles in panel A and the **S** profiles in panel B). The corresponding θ and ϕ values and the C(vinyl)-C(α-acetamide) distances are reported in the Supporting Information. For the **R** profiles, the total activation energy, which is the energy difference between the cyclization transition state [either **(R)-TSa** or **(R)-TSb**] and the lowest-energy state between the reactant and intermediate state [i.e., either **2a**/**2b** or (**proR**)-**2a**/(**proR**)-**2b**] is 20.3 and 22.8 kJ/mol for the cyclization starting from **2a** and **2b**, respectively (panel A of Figure 5). For the **S** profiles, the total activation energy is 31.2 kJ/mol starting from **2a** and 36.9 kJ/mol starting from **2b** (panel B of Figure 5). Hence, the cyclization to the **(R)-3** product displays lower activation barriers (20.3 and 22.8 kJ/mol) than the ones for the cyclization to the **(S)-3** product (31.2 and 36.9 kJ/mol), both starting from the **2a** (20.3 vs 31.2 kJ/mol) and **2b** (22.8 vs 36.9 kJ/mol) structures.

Interestingly, from the energy profiles reported in Figure 5, it can be observed that the cyclization step is strongly irreversible. In fact, in all profiles, the energy difference between radical product **3** and the preceding transition state is higher than 140 kJ/mol. This implies that once the ring closure occurs, its subsequent opening is effectively prohibited. These results clearly show that the selectivity-determining step is indeed a cyclization step.

By reasoning about the stereochemistry of the alkene studied here, in which there are only two substituents in a trans configuration, we can conclude that changing the sign of both the θ and ϕ dihedrals for a given conformation leads to the inversion of the stereocenter, and hence of the potential prochirality, of the molecule. Therefore, a “mirror” scenario is to be expected for the energy profiles starting from the “mirror” conformers with positive θ values (i.e., extracted from the right quadrants of the 2D dihedral distributions): the cyclization to the **(S)-3** product will display lower activation barriers than the ones for the cyclization to the **(R)-3** product starting from both the mirror images of **2a** and **2b**.

### Free Energy Profiles for the Radical Cyclization within GluER-G6

Next, we analyzed the protein effect on the radical cyclization process in GluER-G6 by calculating the corresponding free energy profiles in the presence of the enzyme and solvent by means of the MD-PMM approach (see the Computational and Experimental Methods section). In all pathways, the protein induces a large stabilization of both the prochiral intermediates (either **proR** or **proS**) and the transition states for the cyclization [either **(R)-TS** or **(S)-TS**] with respect to the starting structures. In particular, the intermediate states are stabilized by 30–40 kJ/mol and the transition states by 40–55 kJ/mol. The resulting lowest activation free energies for cyclization in the enzyme are 7.5 and 19.5 kJ/mol for the R- and S-profile, respectively (see insets of Figure 5). These results show that also in the presence of the protein and solvent, the cyclization energy demand is lower for the R enantiomer.

According to the Eyring equation,^41^ a free energy barrier of ≈7–8 kJ/mol corresponds to a cyclization time constant of ≈3–4 ps. This fast timescale is consistent with the experimental time constant of 20 ps measured for the combined cyclization and HAT process.^18^ Notably, according to new MD simulations of the complex in which the ligand was modeled in its exocyclic radical form (intermediate **3**) (see the Supporting Information for the details of these simulations), the most probable C(vinyl)-N5 distance associated with the HAT is approximately 3.5 Å (see the Supporting Information). Such a short distance is compatible with a fast HAT step.

The comparison between the free energy barrier for cyclization in the enzyme and the corresponding energy barrier in the gas phase shows a similar stabilization induced by the protein for both R- and S-products, being ≈10 and ≈12 kJ/mol, respectively (see the insets of Figure 5). This result indicates that, within the errors of the computational procedure used (which is 4 kJ/mol), there is no discernible preferential stabilization of either of the two prochiral transition states.

We further analyzed the PMM-MD results in order to identify the major contributions to the lowering of the R and S cyclization barriers with respect to the corresponding gas-phase energies. In order to achieve this goal, for each protein residue and solvent molecule, the difference of the mean contribution arising from the dipolar term, i.e.,  in eq 1, between the cyclization transition state and the preceding intermediate reported in the inset of Figure 5, was calculated for both the S and R pathways. The results and some more details are reported in Section S1.4 and Figure S3 of the Supporting Information. The residues providing the largest negative contributions, i.e., the largest stabilizing effect on the TS with respect to the preceding intermediate, are the same along both pathways. This result is consistent with the observation reported in the previous paragraph that the stabilization of the barrier by the protein field is similar in both pathways. Interestingly, there is only one residue positioned within the reaction pocket in very close proximity to the substrate, namely, Trp66. All other contributions (Arg61, Arg104, K133, Asp180, and Glu181) originate from charged residues located outside the active-site pocket, the distance from the substrate being in the range of 12–16 Å (see Figure S3 of the Supporting Information).

### Experimental Validation of the Substrate–Enzyme Binding Complex in GluER-G6 and of the Proposed Enantioselective Mechanism

In order to assess the reliability of the binding complex identified in the MD simulations and to analyze the possible role played by the HB between Y177 and the carbonyl group of the substrate, the following mutants were generated by site-directed mutagenesis and their catalytic activity examined, along with the enantioselectivity in the radical cyclization process: G6-A56F, G6-W66A, G6-W100A, and G6-Y177F (see Table 1). According to the binding poses identified in the MD simulation, these four residues form direct interactions with the amide moiety. In particular, the carbonyl is hydrogen bonded to the side chain of Y177; the α-CH2 group is tightly packed within the −CH3 group of A56, the side chain of W100, and the flavin; the *N*-methyl group is less tightly bound, partly interacting with W66 and partly exposed to the solvent.

**Table 1:** 

GluER-type	yield	er
G6	90	97:3
G6-A56F	3	
G6-W66A	63	90:10
G6-W100A	33	88:12
G6-Y177F	79	93:7

The G6-A56F mutant, in which the steric hindrance of the side chain at position 56 is enhanced, showed a negligible yield, suggesting a loss in stability of the binding complex. This is consistent with the finding that the −CH3 group of A56 is in direct contact with the −CH2 group in the computed binding poses: the A56F mutation, which introduces a bulkier group, is indeed expected to have a destabilizing effect. A change in yield, although less dramatic (from 90 to 30%), is observed also for the G6-W100A mutant, which also is a mutation affecting the hydrophobic cavity hosting the α-CH2 group but in the direction of reducing the steric hindrance. For this mutant, only a minor change in the enantioselectivity (88:12 with respect to 94:6) is observed. The relevance of the tight interaction of the α-CH2 group with the active site of the enzyme was further tested by conducting experiments in the G6-enzyme but using an α-methylated substrate, in which a steric hindrance at the same substrate–enzyme interface position is introduced: also, in this case, the measured yield was almost negligible.

Mutation at position 66 (W66A) shows minor changes, with the yield being 60% and the enantioselectivity almost unchanged. These results seem consistent with the computational evidence that the substrate only marginally interacts with residue 66.

**Figure 6:**
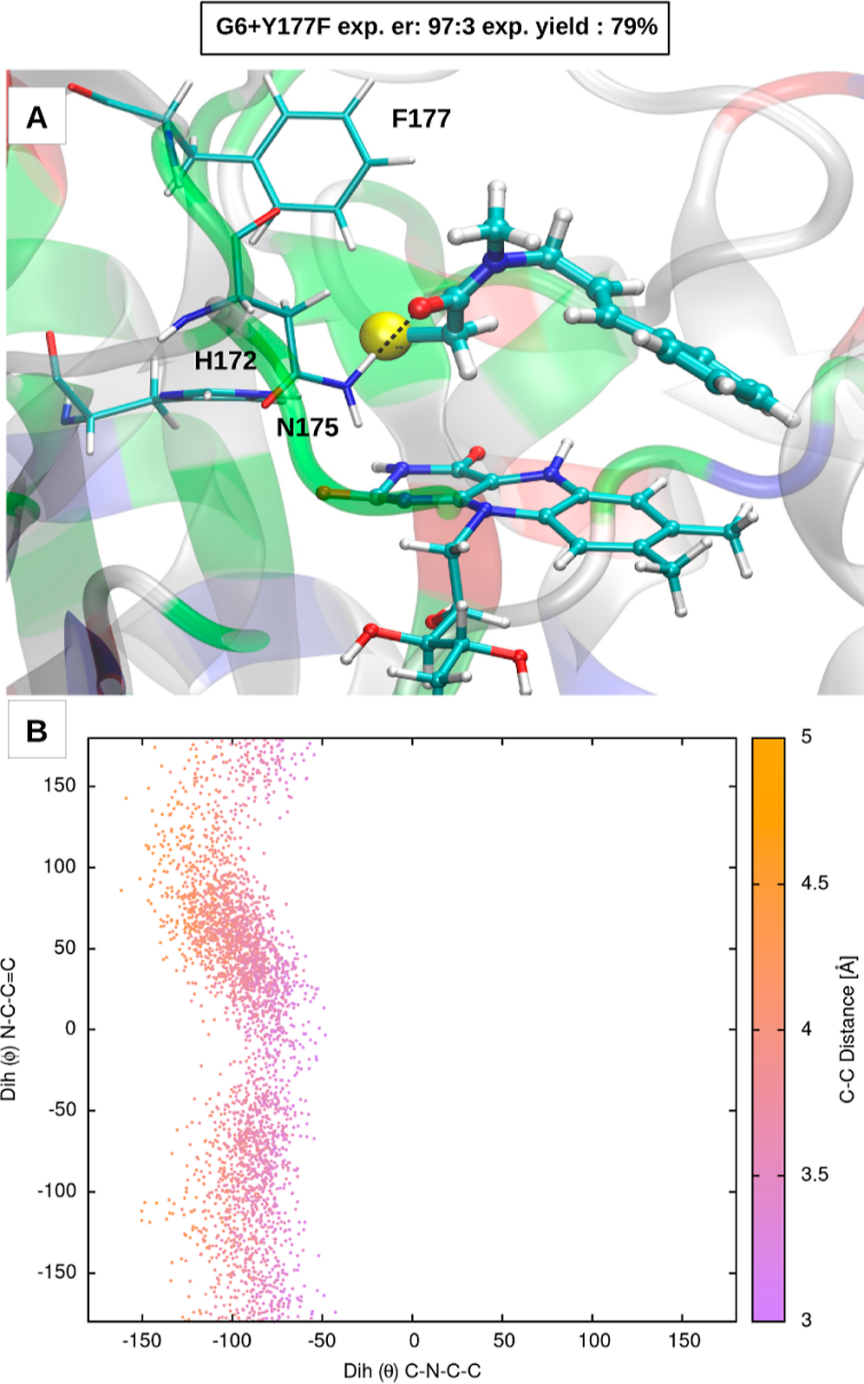
(A) EDA complex in the GluER-G6+Y177F mutant and (B) 2D distribution of the θ and ϕ dihedral angles over the 100 ns long MD trajectory.

Surprisingly, the Y177F mutation, which removes the HB donor to the carbonyl of the substrate, has negligible effects (both the yield and the enantioselectivity were almost unchanged). To rationalize the effect of this mutation, we performed a 100 ns long MD simulation of the G6-Y177F, starting from a representative configuration of the MD simulation of G6. Interestingly, after an initial assessment, a new binding pose was found in which the carbonyl of the substrate forms a HB with the side chain of N175. The analysis of the dihedral angles shows a distribution very similar to that of G6, with only negative θ values being sampled. These results are reported in Figure 6. Given that the experimentally determined enantioselectivity of G6-Y177F is the same as that of G6, our results on simulated G6-Y177F are consistent with our hypothesis that the sign of the θ dihedral angle, which determines the relative orientation of the amide group and the styrene moiety, determines the enantioselectivity.

### Comparison with the OYE1

**Figure 7:**
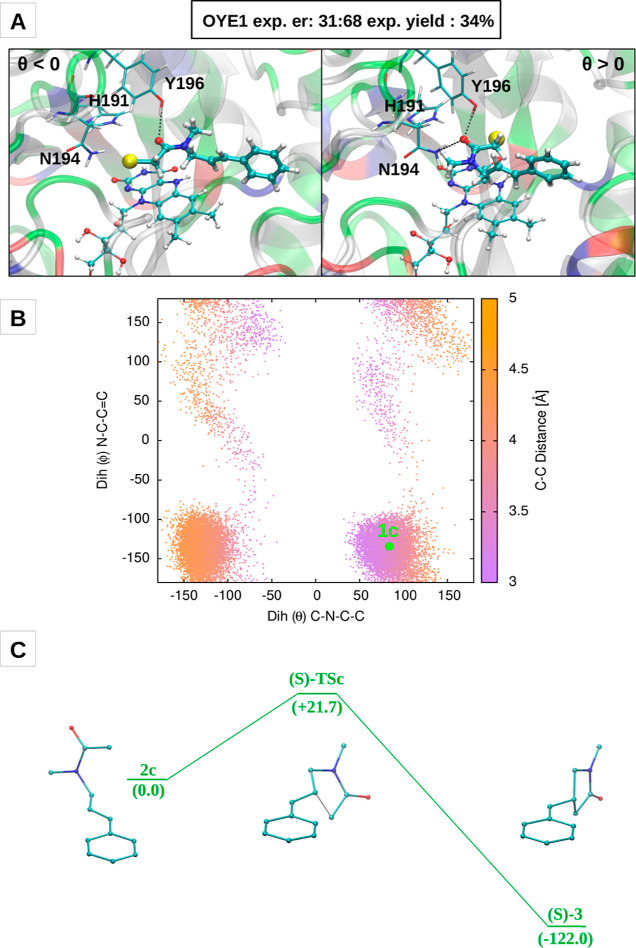
EDA complex and cyclization energy profile for OYE1. (A) Starting configurations of the MD simulations of the EDA complex of the OYE1. (B) 2D distribution of the θ and ϕ dihedral angles over the two MD trajectories. (C) Cyclization energy profile of the **2c** → **(S)-3** pathway.

To rationalize the different enantioselectivity observed experimentally in GluER-G6 and OYE1, we performed two 200 ns long MD simulations of the OYE1 using two different starting configurations of substrate **1** in the active-site cavity, namely, one in which the substrate conformer has a negative θ value and the other in which it has a positive one. Both starting binding poses, which are reported in Figure 7A, were constructed in analogy to representative configurations sampled along the GluER-G6MD simulations. In both cases, the carbonyl of the substrate forms a HB with the side chain of tyrosine 196 (the homolog of Y177 in GluER-G6), but the arrangement within the cavity differs mainly due to a “flip” of the amide group (see Figure 7A).

The analysis of the θ/ϕ dihedral angles reveals that in both trajectories, the sign of the θ value does not change with respect to the initial condition. This behavior is in contrast with that of GluER-G6, where the binding pose with the substrate having a negative θ value was found spontaneously along the MD trajectory and proved to be more favorable than the one with positive θ values. Moreover, the features of the θ/ϕ dihedral distribution calculated over the OYE1 trajectories, reported in Figure 7B, are rather different with respect to GluER-G6. First, a single, instead of two, highly populated basin is observed in the two quadrants at negative θ values and, differently from GluER-G6, the “cyclization” C(vinyl)-C(α-acetamide) distance in the conformers populating this basin is much higher. Second, at positive θ values, the most populated basin is the one on the right-bottom quadrant (i.e., with ϕ values around −135/–150), rather than the one in the middle (i.e., with ϕ values around zero).

We performed gas-phase calculations of the cyclization process to the **(S)-3** product starting from the radical substrate obtained from the centroid of the bottom-right basin of the 2D dihedral distribution, which is indicated as **1c** in Figure 7B. This radical substrate conformer, which we call **2c**, is constructed by means of the same procedure used for the generation of structures **2a** and **2b**, as reported in the Supporting Information. The calculated energy profile, which is reported in Figure 7C, shows an activation energy of 21.7 kJ/mol. This barrier is in line with the activation energies to the **(R)-3** product obtained from the calculations at negative θ values (20.3 and 22.8 kJ/mol) and is thus consistent with the “mirror” scenario introduced above.

In conclusion, our results on OYE1 are consistent with the experimentally determined enantioselectivity of OYE1, which is opposite to the one of GluER-G6 and only in slight favor of the S-enantiomer (er of 31:69). This further supports our hypothesis that the sign of the θ dihedral angle, which determines the relative orientation of the amide group and the styrene moiety, determines the enantioselectivity.

## Conclusions

**Figure 8:**
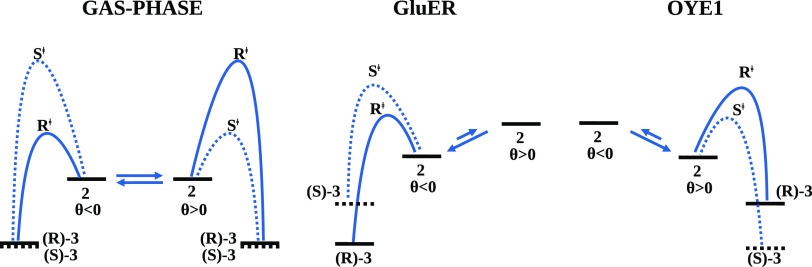
Schematic representation of the enantioselective scenario proposed in the present work for the radical cyclization of an α-chloroamide with EREDs.

In the present work, we address the origin of the opposite enantioselectivity observed experimentally in two different EREDs, namely, GluER-G6 and OYE1, for the photoinduced asymmetric radical cyclization of α-chloroacetamide **1** shown in Figure 1. Our results show that there is no preferential stabilization of either the Si- or Re- π-face of the C=C double bond due to the rapid rotation around the C–C(allyl) bond inside the enzyme, which is due to the lack of bulky substituents of the allylic group. Also, the difference in the TS-height for the R- and S-product formation is independent of the presence of the protein and is, instead, strongly dependent on an intrinsic degree of freedom of the substrate not directly related to the exposure of a given π-face of the double bond, namely, the relative orientation of the amide group with respect to the styrenic group (i.e., the θ dihedral angle shown in Figure 3). Our results show that for negative θ values, the R-TS is lower in energy than the S-TS, while the opposite is true for positive θ values (see the left panel of Figure 8). Hence, if the protein were able to preferentially stabilize substrate conformations with either negative or positive θ values, then an excess of the R-product or S-product, respectively, would be formed; this is indeed the scenario that emerged from our calculations and is schematically depicted in the middle and right panels, respectively, of Figure 8.

As a minor result, our experiments show that by mutating in GluER-G6 tyrosine 177, which is involved in a stable HB with the carbonyl group of the substrate, into a phenylalanine, the yield and enantioselectivity do not change considerably, indicating that this specific HB does not play a crucial role. Nonetheless, our calculations show that once the tyrosine is mutated to a phenylalanine, a backup mechanism takes place and asparagine 175 takes over the role of the tyrosine in stabilizing the binding pose.

In conclusion, the proposed mechanism exhibits the potential for a wider range of applicability. Specifically, it may hold relevance to the general cyclization of alkenes devoid of sterically bulky substituents. Moreover, it could also play a role in cases involving alkenes with a high degree of substitution, thereby contributing to the prevalent π-facial selection mechanism.

## Computational and Experimental Methods

### Molecular Dynamics Simulations

All the MD simulations were carried out with the GROMACS package^42^ and using the CHARMM36 force field^43^ for the enzymes, namely, the ene-reducatases GluER-G6 mutant (PDB:6O08^15^) and OYE1 (PDB:1OYA^44^). The protonation state of the residues was determined by PropKa^45^ in order to reproduce the experimental pH of 8. All the mutations in the GluER were accomplished with the VMD mutation tool.^46^ The parameters for the reduced deprotonated FMN (FMN_*hq*_^–^) and the FMN semiquinone radical (FMN_*sq*_^•^) were taken from a previous parameterization for CHARMM-based force fields.^47^ For the substrate, the parameters were obtained with the CHARMM Web server CgenFF.^48,49^ The simulated system size was selected in order to have a dodecahedron box with a minimum distance of at least 1.2 nm between the protein and the box. The water molecules were described with the TIP3P model,^50^ and an appropriate number of counterions were added to achieve charge neutrality of the entire system. The Particle Mesh Ewald^51^ method was used for the long-range electrostatic interactions applying a 0.132 Fourier spacing and a 1.1 nm cutoff. For each system, after energy minimization, the desired temperature of 298 K was reached by means of an annealing protocol from 50 to 298 K with a 100 ps long MD trajectory. Then, production trajectories were carried out in the *NPT* ensemble (i.e., at constant temperature, pressure, and number of atoms). A time step of 0.002 ps was employed in conjunction with the LINCS algorithm^52^ to constrain bond lengths involving hydrogen atoms. The velocity rescaling temperature coupling^53^ (with τ_T_ = 0.002 ps) was used to maintain the temperature constant and the Berendsen barostat (with τ_P_ = 1.0 ps) for the pressure coupling.^54^

### Quantum Mechanics and Minimum Energy Path Calculations

All the quantum chemical calculations were performed using the ORCA package^55^ with the density functional theory level of theory^56^ applying the B3LYP functional^57^ and employing the 6-31G* basis set.^58^ Based on the conformational analysis of the substrate within the reaction cavity of GluER-G6 and OYE1 as presented in the Results section, three initial structures of the radical substrate were generated for the calculation of radical cyclization energy profiles. These structures, labeled **2a**, **2b**, and **2c**, were then optimized in the gas phase while constraining the two dihedral angles, θ and ϕ, as detailed in the Results section. This constraint was applied to account for the excluded volume of the enzyme’s active site. Then, the structures of the chiral products of the cyclization, termed **(S)-3** and **(R)-3**, were obtained by relaxation with a shortened cyclization C–C distance. The optimized C–C distance was 1.54 Å for both the enantiomers. The cyclization internal energy variation for the paths connecting the starting radical substrates and the products **(R)-3** and **(S)-3** were calculated with the NEB^59^ method with the climbing image (CI)^60^ approach as implemented in ORCA. Based on previous calculations,^61,62^ in the present work, 10 images were used, including the starting and final points. The convergence criteria on the energy variation along the NEB calculations were set to “tight” (maximum perpendicular force to the reaction coordinate: 5.0^–4^ Ha/Bohr; maximum force RMS: 2.0^–4^ Ha/Bohr).

### Perturbed Matrix Method for Reaction Free Energy Calculation

In order to estimate the reaction free energy, i.e., the free energy variation along a reaction coordinate, we utilized the Perturbed matrix method associated with molecular dynamics simulation (hereafter MD-PMM). MD-PMM, the details of which are reported elsewhere,^30,63^ is a theoretical–computational perturbative approach that allows the reconstruction of the ensemble of perturbed electronic eigenstates of a subportion of a MD box, hereafter termed a quantum center (QC), in the presence of the electrostatic (semiclassical) interaction with the rest of the simulated system (e.g., the solvent and the protein). Briefly, a set of unperturbed (gas-phase) QC eigenstates (ϕ_*j*_^0^) are first calculated. Subsequently, these states are used as the basis set to represent the QC perturbed operator
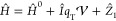
1
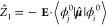
2where  is the unperturbed Hamiltonian matrix (i.e., constructed with the unperturbed QC eigenstates), *Î* is the identity matrix, *q*_T_ is the total QC charge,  is the electric potential exerted by the environment onto the QC of mass, **μ̂** is the electric dipole operator, and **E** is the electric field produced by the atomic–molecular environment onto the QC of mass at each frame of the trajectory. Diagonalization of the matrix provided above provides, at each frame of the MD simulation, a set of eigenvectors and eigenvalues representing the QC perturbed properties. If we recalculate the basis set (ϕ_*j*_^0^) along a predefined reaction coordinate, we can then obtain, at each frame of the MD simulation, a collection of perturbed electronic eigenvalues (*U*) as a function of the position of the QC along the reaction coordinate. Hence, we can calculate the (standard) free energy as a function of the reaction coordinate by means of the equation^31,64,65^
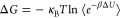
3where κ_B_ is the Boltzmann constant and Δ*U* is the difference between the perturbed energies between a point of the reaction coordinate and the initial position evaluated. The bracket indicates that the average is performed within the ensemble (i.e., the MD simulation) of the QC in the initial position of the reaction coordinate. For using the above method, we evaluated the unperturbed eigenstates of the selected QCs [**2a**, **2b**, and **2c** for the starting points; **(proR)-2a**, **(proR)-2b**, and **(proS)-2** for the intermediate states; **TSa**, **TSb**, **TS’b**, **(R)-TSa**, **(R)-TSb**, **(S)-TSa**, **(S)-TSb**, and **(S)-TSc** for the transition states; and **(R)-3** and **(S)-3** for the final states]. Thus, five free energy variations of the five gas-phase cyclization pathways are obtained. In the Results section, for each path, only the free energy difference between the lowest-energy intermediate and the highest transition state is reported.

### Photoenzymatic Experiments

The synthesis and characterization of the model chloroamide substrate, lactam product, and lactam racemate are detailed in Biegasiewicz et al.^15^ The general procedure for the photoenzymatic experiments and analysis was adapted from Page et al.^25^ All reactions were performed in an anaerobic chamber. Reactions were run with 20 μmol of the α-chloroamide substrate (see Figure 1). A shell vial with a magnetic cross stir bar was charged with 240 μL of “turnover mix” [GDH-105 (5 mg/mL), glucose (40 mg/mL), and NADP^+^ (1.5 mg/mL) in KPi 100 mM, pH 8]. An additional 100 mM KPi (pH 8) was added such that the final reaction volume was 620 μL. Next, 2 mol % of the enzyme was added. Finally, 20 μL of a stock solution of the chloroamide substrate in (isopropyl alcohol) IPA (20 μmol/20 μL) was added to the shell vial. The vial was sealed with a rubber septum and brought out of the anaerobic chamber where it was placed on a stir plate at 400 rpm under a fan and irradiated with cyan LEDs (50 W Chanzon high-power LED chip, λ_max_ = 490 nm, measured photon flux = 12,000 mM/m^2^ s) for 48 h. The product yield was determined using product standard curves of liquid chromatography–mass spectrometry. Additional information about the HPLC procedure and corresponding curves are reported in Section S2 of the Supporting Information.

## Supporting Information Available

MD simulations of the radical substrate 2 in complex with GluER-G6, analysis of the gas-phase optimization of intermediate **2**, structural details of the different states sampled along the cyclization pathways, contribution of protein residues to the stabilization of the cyclization barrier, MD simulations of the exocyclic (R)-**3** radical in complex with GluER-G6, and photoenzymatic reaction yield and selectivity analysis (PDF)
