# Supplementary material for: Evidence of a Distinctive Enantioselective Binding Mode for the Photoinduced Radical Cyclization of α-Chloroamides in Ene-Reductases
Source: ACS Catal. 2023 Nov 10;13(23):15310–21. doi: 10.1021/acscatal.3c03934 (PMC10696551; doi:10.1021/acscatal.3c03934)
Supplement: Supplementary file 1 — cs3c03934_si_001.pdf [file cs3c03934_si_001.pdf]

Supporting Information:
Evidence of a distinctive enantioselective binding mode for the photo-induced radical cyclization of α-chloroamides in ene-reductases

Matteo Capone,[†,§] Gianluca Dell'Orletta,[†,§] Bryce T. Nicholls,[‡] Gregory D. Scholes,[¶] Todd K. Hyster,[‡] Massimiliano Aschi,[†] and Isabella Daidone[*,†]
E-mail: isabella.daidone@univaq.it

† Department of Physical and Chemical Sciences, University of L'Aquila, via Vetoio (Coppito 1), 67010 L'Aquila, Italy
‡ Department of Chemistry and Chemical Biology, Cornell University, Ithaca, New York, 14853, United States
¶ Department of Chemistry, Frick Laboratory, Princeton University, Princeton, New Jersey, 08544, United States
§These authors contributed equally

Contents

1. **Computational Supplementary Information**
   1.1 MD simulations of the radical substrate **2** in complex with GluER-G6
   1.2 Analysis of the gas-phase optimization of intermediate **2**
   1.3 Structural details of the different states sampled along the cyclization pathways
   1.4 Contribution of protein residues to the stabilization of the cyclization barrier
   1.5 MD simulations of the exocyclic **(R)-3** radical in complex with GluER-G6
2. **Experimental Methods**
   2.1 Photoenzymatic reaction yield and selectivity analysis
3. **References**

1. Computational Supplementary Information

1.1 MD simulations of the radical substrate 2 in complex with GluER-G6

Molecular Dynamics (MD) simulations of the radical substrate **2** in complex with GluER-G6 were performed according to the following procedure: (i) 60 configurations were extracted from the MD trajectories of the complex between substrate **1** and GluER-G6; (ii) for each configuration of the complex, the Cl anion was removed and the resulting configurations were energy-minimized using a modified version of the force field of the ligand in order to describe the radical substrate **2** (see below); the FMN cofactor was treated in its semiquinone form (FMN_{sq}) using the parametrization provided in ref [1]; (iii) each minimized configuration was then simulated for 50 ps in the same conditions of the original complex (see Methods section of the manuscript). This time interval was chosen on the basis of the experimental spectroscopic measurements according to which the average reaction time of the full reaction (including, hence, also the hydrogen atom transfer following the cyclization) is of the order of 20 ps.

For the partial charges of the substrate **2** radical intermediate the RESP charges calculated with Gaussian 16 [2] were used. Given that the radical was mostly localized on the aromatic ring, the α-acetamide carbon and its bonded hydrogens were described using the terminal CH_2 atom types in CHARMM. In order to maintain the planarity of the CH_2 group with respect to the carbonyl group, as observed in the quantum-mechanics optimization of the substrate **2** radical intermediate, an improper dihedral involving the H-C-C-O atoms was added with a constant of 1000 kj/mol*rad, in analogy with other planar carbonyl-related groups.

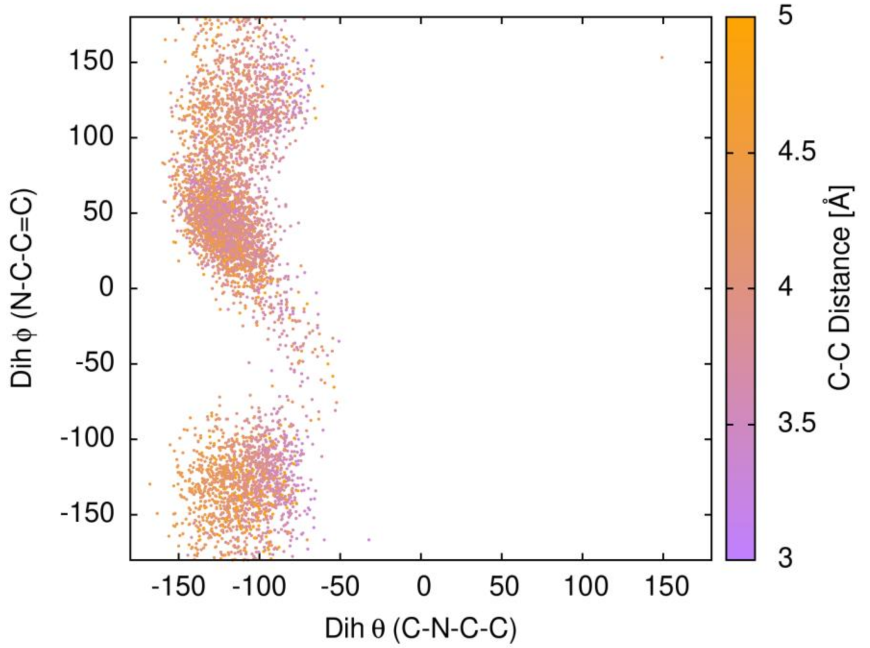

Figure S1. 2D distribution of the θ and ϕ dihedral angles over the MD simulations of the substrate **2** radical intermediate within GluER-G6.

To assess the feasibility of the dissociation of the chloride upon the mesolytic cleavage step, the distance between the Cl^- anion and the C(α-acetamide) of the ligand along the MD trajectories was analyzed. The analysis shows that there is room in the active site for

the detachment of the Cl^- anion and that in many of the trajectories the ion moves to a second coordination shell (~8 Å) before leaving the active-site cavity in tens of picoseconds. Four examples of these trajectories are given in Figure S2.

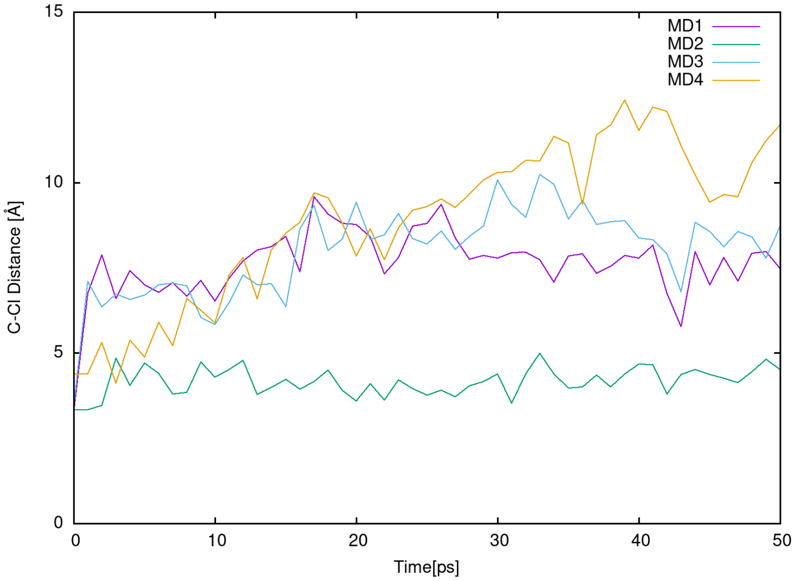

Figure S2: Examples of the time-dependence of the C(α-acetamide) - Cl^- distance along four 50 ps-long MD trajectories of the complex between the GluER enzyme and the dechlorinated radical form of the substrate (i.e. intermediate **2**).

1.2 Analysis of the gas-phase optimization of intermediate 2

In addition to the constrained quantum-mechanical (QM) gas-phase optimization presented in the manuscript of the reactant structures **2a** and **2b**, we also performed unconstrained optimizations for comparison. The calculations show that for **2b** the unconstrained optimization results in a very similar structure to that obtained with constraints. In fact, the values of the dihedral angles θ and ϕ are nearly identical: θ= -100.00/ϕ= -125.00 for the constrained structure and θ= -99.50/ϕ= -120.90 for the unconstrained structure. Furthermore, the unconstrained structure exhibits the same stability as the constrained one, being the unconstrained optimized-structure lower in energy by only 0.08 kJ/mol. Conversely, the unconstrained optimization for **2a** yielded a different structure from that obtained with constraints (θ= -120.00/ϕ= 48.00) but very similar to that of the intermediate **(proS)-2**, as shown by comparing the dihedral angles θ and ϕ: θ= -88.20/ϕ= 2.71 for the intermediate **(proS)-2** and θ= -95.33/ϕ= 6.94 for the unconstrained optimized-structure **2a**. Concerning the energy, the unconstrained structure is 9.24 kJ/mol more stable than the constrained one, a difference in energy equal to that between **2a** and **(proS)-2**, which is 9.30 kJ/mol. Therefore, in the absence of the protein, the centroid of the **2a** basin would be shifted closer to the **proS-2** intermediate.

As the unconstrained **2b** structure is almost identical to the constrained one, we don't expect any change in the cyclization profiles starting from this structure. Concerning the profiles starting from **2a**, as the unconstrained **2a** structure is similar to **(proS)-2**, we don't expect significant differences in the cyclization energy barrier that leads to **(S)-3**. On the other hand, a higher cyclization barrier might be expected for the profile leading to **(R)-3** because of the opposite prochirality of the **(proS)-2** intermediate. In fact, an additional structural rearrangement would be necessary in order to achieve the R-prochirality.

1.3 Structural details of the different states sampled along the cyclization pathways

2a → (R)-3				
	**θ**	**ϕ**	**C-C (Å)**	**ΔE (kJ/mol)**
**2a**	-120.00	48.00	3.93	0.00
**TSa**	-89.02	102.05	3.63	18.61
**proR-2a**	-47.96	146.19	2.94	3.78
**(R)-TSa**	-36.52	153.98	2.46	20.29
**(R)-3**	-20.51	152.39	1.55	-128.90
**2b → (R)-3**				
	**θ**	**ϕ**	**C-C (Å)**	**ΔE (kJ/mol)**
**2b**	-100.00	-125.00	3.69	0.00
**TSb**	-78.14	167.70	3.31	7.67
**proR-2b**	-70.85	149.60	3.20	5.80
**(R)-TSb**	-32.85	150.78	2.40	22.84
**(R)-3**	-20.51	152.39	1.55	-120.33
**2a → (S)-3**				
	**θ**	**ϕ**	**C-C (Å)**	**ΔE (kJ/mol)**
**2a**	-120.00	48.00	3.93	0.00
**proS-2**	-88.20	2.71	3.43	-8.17
**(S)-TSa**	-31.80	-74.74	2.41	23.00
**(S)-3**	-19.64	-93.55	1.55	-124.04
**2b → (S)-3**				
	**θ**	**ϕ**	**C-C (Å)**	**ΔE (kJ/mol)**
**2b**	-100.00	-125.00	3.69	0.00
**TS’b**	-74.20	-72.98	3.33	29.93
**proS-2**	-83.14	2.19	3.36	1.85
**(S)-TSb**	-39.65	-61.37	2.37	36.92
**(S)-3**	-19.63	-93.55	1.55	-115.56
**2c → (S)-3**				
	**θ**	**ϕ**	**C-C (Å)**	**ΔE (kJ/mol)**
**2c**	83.78	-133.96	3.41	0.00
**(S)-TSc**	32.97	-149.65	2.37	21.67
**(S)-3**	20.51	-152.39	1.55	-122.00

Table S1. Energies, θ and ϕ values and C(vinyl)-C(α-acetamide) distances for the reactants, possible intermediates, transition states and products along the following cyclization pathways: **2a** -> **(R)-3** and **2b** -> **(R)-3** (the **R** profiles) and **2a** -> **(S)-3** and **2b** -> **(S)-3** (the **S** profiles). The energies of each path are always reported with respect to the corresponding first state; note that structure **2b** is 8.50 kJ/mol more stable than structure **2a.**

1.4 Contribution of protein residues to the stabilization of the cyclization barrier

We further analyzed the Perturbed Matrix Method (PMM) results in order to identify the major contributions to the lowering of the **R** and **S** cyclization barriers. Examples of this type of analysis applied to other observables, such as redox potential shifts or vibrational frequency shift, can be found in references [3] and [4]. For each residue and solvent molecules, we calculated the difference of the mean energy contribution arising from the dipolar term, i.e. $\hat{Z}_1$ in eq. 1 of the main text of the manuscript, between the cyclization transition state and the preceding intermediate (i.e. the states reported in the inset of Figure 5 of the main text). In Figure S3 we report the results for the residues in the range 50-200, which is the range in which the highest contributions are observed. The residues providing the largest negative contributions, i.e. the largest stabilizing effect on the TS with respect to the intermediate, are highlighted in the figure and are essentially identical in both pathways. This confirms that the protein field does not play a key role in fine-tuning the relative energy of the transition state of the **S** and **R** pathways.

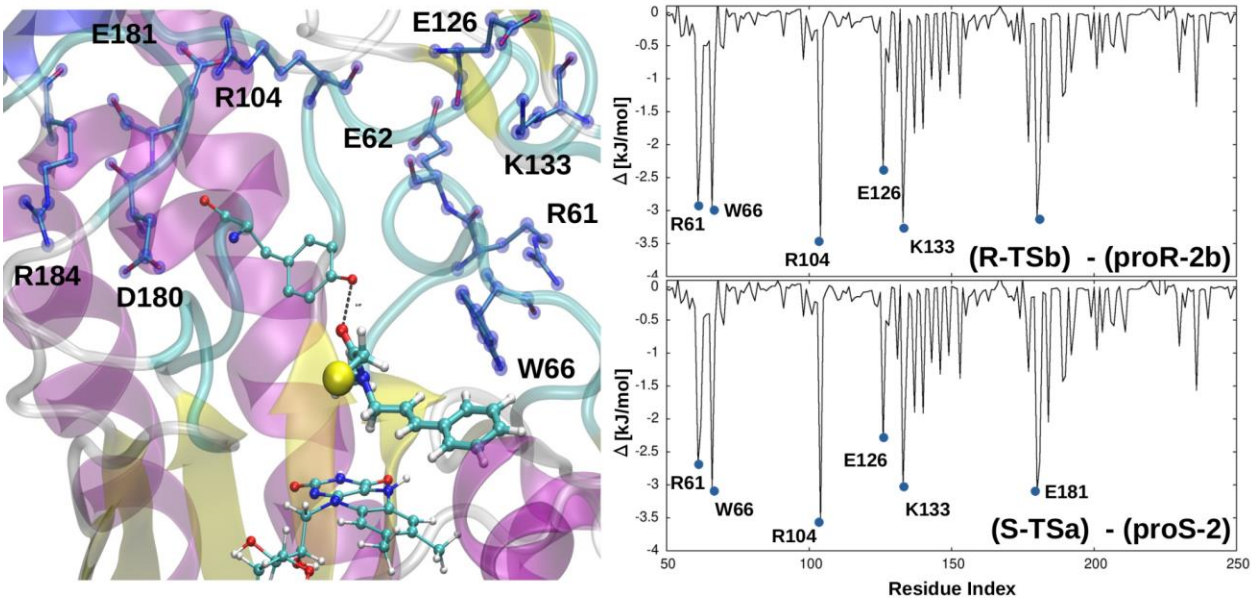

Figure S3: **Δ** = < $\boldsymbol{\mu}_{TS}$ **E**> - <$\boldsymbol{\mu}_{int}$ **E**>, i.e. the difference between the mean dipolar contribution along the MD trajectory to the energy of the transition state (**R-TSb** or **S-TSa**) and the intermediate state (**proR-2b** or **proS-2**), is reported for residues 50 to 200, which is the range in which the highest contributions are observed. The left panel highlights the residues providing the most relevant contributions.

1.5 MD simulations of the exocyclic (R)-3 radical in complex with GluER-G6

Ten 50-ps long MD simulations of the exocyclic **(R)-3** radical within the active-site of GluER-G6 were performed. Most of the force field parameters for **(R)-3** were derived, as for substrate **1,** with the CgenFF web server [5,6] and using the RESP charges calculated with Gussain 16 [2]. For the vinylic carbon atom we imposed a sp2 hybridization utilizing the same atom type used for the same atom in the intermediate **2**. This choice was based on the QM-optimized structure of the **(R)-3** radical which revealed the delocalization of the radical over the styrene moiety, resulting in a nearly planar, sp2-like geometry of the vinylic carbon.

The distribution of the distance between the C(vinyl) atom of the exocyclic **(R)-3** radical and the N5 atom of FMN_{sq}, i.e. the distance associated to the two heavy atoms that exchange the hydrogen atom, along the MD simulations is reported in Figure S4.

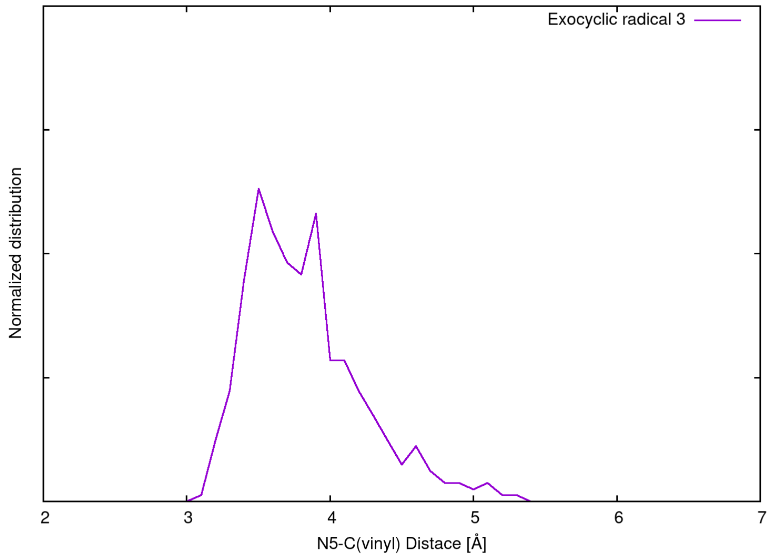

Fig. S4: Distribution of the N5(FMN_{sq}) - C(vinyl)(substrate) distance along the MD simulations of the exocyclic intermediate radical **3** in complex with GluER-G6.

2. Experimental Methods

2.1 Photoenzymatic reaction yield and selectivity analysis

All sample vials contain a final volume of 1.0 mL MeCN and include 100 μL of a 5 mg/mL solution of 1,3,5-tribromobenzene standard in MeCN. The x-axis measurement is the ratio of the Liquid chromatography–mass spectrometry (LCMS) peak areas of the compound of interest to the standard. LCMS method: LCMS_30_95_10M.

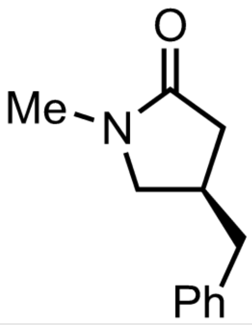

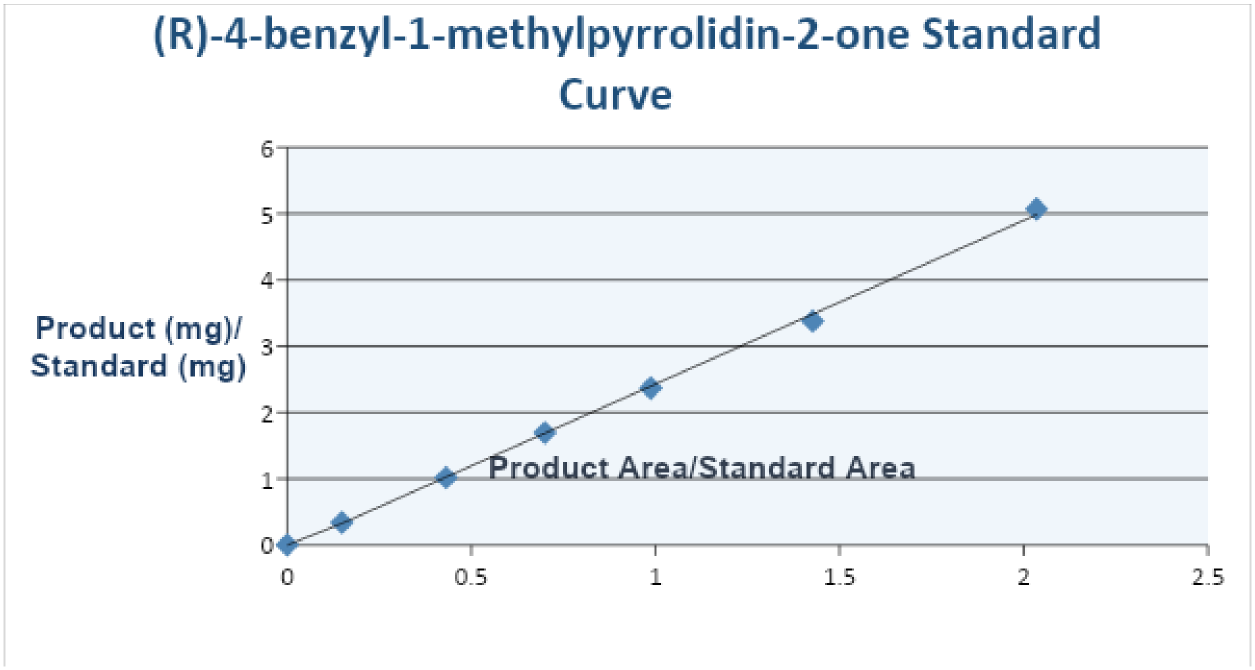

Figure S5. (R)-4-benzyl-1-methylpyrrolidin-2-one (i.e. the reaction product) HPLC analysis. Method Used: AS-20IPA-45M.

Racemate

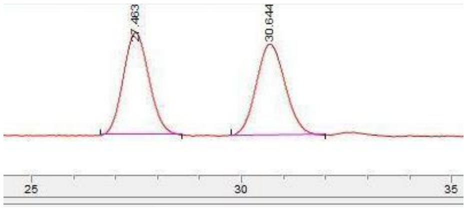

#	Time	Type	Area	Height	Width	Area%	Symmetry
1	27.463	BB	1017.5	23.7	0.5387	50.281	0.941
2	30.644	BB	1006.1	21	0.5872	49.719	0.809

G6-W66A

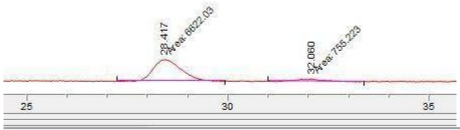

#	Time	Type	Area	Height	Width	Area%	Symmetry
1	28.417	MM	6622	131.9	0.837	89.763	0.705
2	32.06	MM	755.2	15.9	0.7934	10.237	1.259

G6-W100A

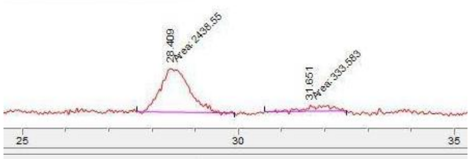

#	Time	Type	Area	Height	Width	Area%	Symmetry
1	28.409	MM	2438.6	51.4	0.7905	87.967	0.548
2	31.651	MM	333.6	7.6	0.7325	12.033	0.476

G6-Y177F

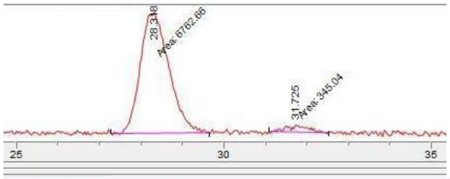

#	Time	Type	Area	Height	Width	Area%	Symmetry
1	28.318	MM	6762.7	141.5	0.7967	95.146	0.92
2	31.725	MM	345	9.1	0.6347	4.854	0.717

3. References

(1) Aleksandrov, A. A molecular mechanics model for flavins. J. Comp. Chem. 2019, 40, 2834–2842

(2) Frisch, MJ ea, et al. Gaussian 16, revision C. 01. 2016.

(3) Zanetti-Polzi, L., Bortolotti, C. A., Daidone, I., Aschi, M., Amadei, A., & Corni, S. 2015. A few key residues determine the high redox potential shift in azurin mutants. Org. Biomol. Chem. 13(45), 11003-11013.

(4) Amadei, A., & Aschi, M. Stationary and Time-Dependent Carbon Monoxide Stretching Mode Features in Carboxy Myoglobin: A Theoretical–Computational Reappraisal. J. Phys. Chem.B, 2021, 125(50), 13624-13634.

(5) Anommeslaeghe, K.; Hatcher, E.; Acharya, C.; Kundu, S.; Zhong, S.; Shim, J.; Darian, E.; Guvench, O.; Lopes, P.; Vorobyov, I.; MacKerell Jr, A. D. CHARMM general force field: A force field for drug-like molecules compatible with the CHARMM all-atom additive biological force fields. J. Comp. Chem. 2010, 31, 671–690

(6) Gutiérrez, I. S.; Lin, F.-Y.; Vanommeslaeghe, K.; Lemkul, J. A.; Armacost, K. A.; Brooks III, C. L.; MacKerell Jr, A. D. Parametrization of halogen bonds in the CHARMM general force field: Improved treatment of ligand–protein interactions. Bioorg. Med. Chem 2016, 24, 4812–4825.
